# Holistic Medicine IV: Principles of Existential Holistic Group Therapy and the Holistic Process of Healing in a Group Setting

**DOI:** 10.1100/tsw.2003.124

**Published:** 2003-12-23

**Authors:** Soren Ventegodt, Niels Jorgen Andersen, Joav Merrick

**Affiliations:** The Quality of Life Research Center, Copenhagen K, Denmark

**Keywords:** quality of life, QOL, philosophy, human development, holistic medicine, public health, group therapy, Denmark

## Abstract

In existential holistic group therapy, the whole person heals in accordance with the holistic process theory and the life mission theory. Existential group psychotherapy addresses the emotional aspect of the human mind related to death, freedom, isolation, and meaninglessness, while existential holistic group therapy addresses the state of the person's wholeness. This includes the body, the person's philosophy of life, and often also love, purpose of life, and the spiritual dimension, to the same extent as it addresses the emotional psyche and sexuality, and it is thus much broader than traditional psychotherapy.Where existential psychotherapy is rather depressing concerning the fundamental human condition, existential holistic therapy conceives life to be basically good. The fundamentals in existential holistic therapy are that everybody has the potential for healing themselves to become loving, joyful, sexually attractive, strong, and gifted, which is a message that most patients welcome. While the patient is suffering and fighting to get through life, the most important job for the holistic therapist is to keep a positive perspective of life. In accordance with these fundamentals, many participants in holistic group therapy will have positive emotional experiences, often of an unknown intensity, and these experiences appear to transform their lives within only a few days or weeks of therapy.An important idea of the course is Bohm's concept of “holo-movement” in the group, resulting from intense coherence between the group members. When the group comes together, the individual will be linked to the totality and the great movement forward towards love, consciousness, and happiness will happen collectively — if it happens at all. This gives the individual the feeling that everything that happens is right, important, and valuable for all the participants at the same time. Native Americans and other premodern people refer to this experience as “the spiritual design”. This design is actually an underlying regulation that appears when people, through their feelings and engagement for each other, tie the group together and engage their complex emotional intelligence. Practically, this means that all participants are sunk in the same information matrix, so that everybody learns from each other. Everything that happens in the perception of each trainee has immediate and developing relevance for him.Spontaneous healing happens far more effectively in a group setting, where all the participants stand together and support each other, than it does in the clinic, where the therapist is alone with the patient. A 5-day course in personal development can be compatible to a half year of holistic individual therapy.

### INTRODUCTION

While group psychotherapy has been an established practice for years, existential holistic group therapy has often been regarded as more difficult[1]. Psychotherapy often deals with aspects of mind and emotion, while existential theory deals with aspects of the wholeness of the human being (the soul). The holistic process theory[2] explains the general principle of existential healing, whether this is done in a group setting or in clinical practice on an individual basis.

While this process takes great effort and experience to bring about in individual treatment, our experience tells us that it is not as difficult to make holistic healing processes work in a group setting. We believe that a lot can be gained by doing so.

The amount of time in which the patient is “in process” under a normal session of existential therapy is maybe only 10 or 15 minutes, summing up to approximately 3 to 6 hours during a half year of therapy. In a 5-day course with intensive existential group therapy, a participant can achieve 10 or even 20 hours of process time. This explains how a 5-day course of personal development can provide a better result than a half year of individual, holistic therapy.

Much of the experience gained with existential holistic group therapy is difficult to put in a formula, as it is about the quality of the therapeutic space and the spirit of the intervention. The procedure wins a lot in efficiency by being informal, experimental, and fluent so the participants know so little about what to expect that they let go of their control of the situation. These aspects are difficult to map and explain, but intuitively are fair and reasonable. The participants are more willing to assume responsibility if they are described as trainees or participants rather than patients. The therapists are more likely to be listened to if they assume the role as teachers of life philosophy (respected like a kind of zen-master), rather than insisting on their professional titles or roles as physicians, nurses, or other kind of care personnel. Below are only listed the issues which more easily come under scientific scrutiny.

### EXISTENTIAL HOLISTIC THERAPY AND EXISTENTIAL PSYCHOTHERAPY ARE VERY DIFFERENT

The domain of existential holistic therapy is the whole person, the holos, the soul, the deep self, higher self, real self, or simply the self, in contrast to the ego, false self, or illusory self. Existential psychotherapy[1] addresses the emotional aspect of the human mind related to death, freedom, isolation, and meaninglessness, while existential holistic therapy addresses the state of the persons *wholeness*[2]. Existential holistic therapy is not psychotherapy, as it addresses the body, the person’s philosophy, and the spirit, to the same extent as it addresses the psyche, normally translated in practice to something like “the emotional mind”.

Contrary to the normal practice of existential psychotherapy, the development of a verbal consciousness and a sound personal philosophy is very important to existential, holistic therapy. Existential holistic therapy is not based on Freud's concept of the sexual urge or the like, but on the concept of the purpose of life, which is embedded in the wholeness of the human being and not in any part of it[3]. Existential holistic therapy makes an equal point on holding and processing[2] and the belief that the healing of the human existence at large improves both health, quality of life, and the ability to function[4,5,6]. Contrary to neo-Freudian (interpersonal) psychodynamic theory[7,8,9], in existential holistic therapy it is assumed that the human being is a product of his or her intentions[10] and, in particular, his or her original purpose of life and denial of this purpose[11,12,13,14,15]. The holistic process theory is strongly inspired by Jung's theory of the ego, the self, and the shadow[12,16]. Existential, holistic therapy can be carried out in an individual[2,16,17] or group setting[18,19,20,21].

Where existential psychotherapy is rather depressive concerning the fundamental human conditions — life is basically meaningless, everybody is basically isolated, freedom is basically empty, and life is mostly a relentless enterprise ending up with death — existential holistic therapy is fundamentally positive. It looks at life as basically meaningful, joyful, powerful, wise, and coherent. This makes holistic therapy very different, and the feel of existential holistic therapy is much more "naive and innocent" than the somewhat "cynical and experienced" feel of existential psychotherapy.

It is true we are free, but freedom is not emptiness as it is our ticket to autonomy — what makes it possible for us to be our self with our own consciousness, perspective, individuality, and purpose of life. And it is true that we shall die in the end, but we can die peacefully, happily, and saturated of days, only if we lived out our purpose. The belief in existential holistic therapy is that everybody has the potential for healing and being themselves, loving, strong, joyful, sexual, and gifted, a message that is most welcome with patients. While the patient is suffering and fighting to get order in his or her life, the most important job for the holistic therapist is to keep a positive perspective of life.

The positive perspective allows the patients to open up to hidden reservoirs of energy and pleasure, interests, and intelligence. In accordance with this, many patients, opening up to their negative feelings and existential pains, also experience highly unexpected strong, positive feelings, often of unknown intensity. The reason for this is, of course, that both the negative and positive feelings are repressed at the same time. These intense emotional experiences — invited by holistic existential therapy based on quality of life philosophy[10,22,23,24,25,26,27], quality of life theory[4,5,6], and existential theory[3,11,12,13,14,15] — appear to transform the patients' comprehension of life and existence sometimes within only a few days or months of therapy[19,20,21].

### THE LIFE MISSION THEORY AND THE HOLISTIC PROCESS THEORY OF HEALING

The life mission theory[3,11,12,13,14,15] states that everybody has a purpose of life, or huge talent. Happiness comes from living this purpose and succeeding in expressing the core talent in one's life. To do this, it is important to develop as a person into what is known as the natural condition, a condition where the person will know himself and use all his efforts to achieve what is most important for him. The holistic process theory of healing[2] states that the return to the natural state of being is possible, whenever the person gets the resources needed for existential healing. The resources needed are in the dimensions awareness, respect, care, acknowledgment, and acceptance with support and processing in the dimensions feeling, understanding, and letting go of negative attitudes and beliefs. The preconditions for holistic healing are trust and the intention for the healing to take place. Existential healing is not a local healing of any tissue, but a healing of the wholeness of the person, making him much more resourceful, loving, and knowledgeable of himself and his own needs and wishes. In letting go of negative attitudes and beliefs, the person returns to a more responsible existential position and an improved quality of life. The philosophical change of the person in healing is often a change towards preferring difficult problems and challenges, instead of avoiding difficulties in life. The person who becomes happier and more resourceful often also becomes more healthy, more talented, and able to function.

### THE PHILOSOPHICAL, SPIRITUAL, AND PRACTICAL FRAME OF THE COURSE

There are a series of important steps that have to be fulfilled before the trainee feels that the course has been successful and of value. Independently from this, everything that happens can be considered occasions that potentially can give insight and learning, when the trainee gets an ability to reflect upon the events and reflect on his part of the responsibility of what has happened. It is emphasized from the start that the trainee is fully responsible for himself and his actions under the whole course. Thus it is not psychotherapy or medical psychiatric treatment in any traditional sense, but rather a convention of personal development as the main goal, with some exact rules that the trainee has to accept, if he wants to take part in the course.

In accordance with the underlying theories[2,3,4,5,6,11,12,13,14,15], the idea behind the course is to obtain proximity of trust and love, so that the trainees can receive holding and support from each other. It is essential to highlight the major importance of an unconditional loving, positive, and trustful attitude from the leaders of the course, in order to provide the necessary intimacy, openness, and closeness between the participants. Thus, everything that happens with the trainee must, without any exceptions, be considered as something good and necessary for him — this includes any failure that may come across from the partner, other trainees, or even the teacher. The teachers must constantly show their good intentions of helping the trainees and must scrutinize themselves for any impure or egotistic motive and always have to consider the occurrence of their own projections on the trainees, whenever something gets unpleasant or provoking during the course.

The course is teaching philosophy of life as described elsewhere[10,22,23,24,25,26,27,28]. This is done so that the mind of the trainees shall support the development process, instead of fighting it. Words, concepts, theories, and models have very little to do with the holistic process of healing, which most commonly takes place in a context without words or emotions and is carried out initiated by the trainees' intention of creating a better life. Nevertheless it is important that the mind has a permitting and accepting role. If the mind of the trainee is negatively disposed to the course or parts of it (like, for example, a teacher, a principal, or some practical thing), this can obstruct the development process in a serious way [29,30].

An important component of the course is the holo-movement in the group[31], resulting from intense coherence between the group members[25]. When the group is together, the individual will be linked to the totality and the great movement forward toward love, consciousness, and happiness will happen collectively — if it happens at all. This gives the individual the feeling that everything that happens is right, important, and valuable for all the participants at the same time. Native Americans and other premodern people refer to this experience as "the spiritual design". This design is actually an underlying regulation that appears when people, through their feelings and engagement for each other, tie the group together and engage their complex emotional intelligence. Practically, this means that all participants are sunk in the same information matrix, so that everybody learns from each other. The experienced therapist or teacher gets a very surprising sense for the trainees' ability to relax, feel good, let go of any personal reservation or negative attitude, and let his existence be part of the course and be a part of the group and the holo-movement. Everything that happens in the perception of each trainee has immediate and developing relevance for him.

The premise for the course does not have to be fashionable, but it has to be aesthetic and give enough isolation from the surrounding world so that all feelings can be expressed freely, without having any conflict with people outside the course. The food must be good and plentiful, and any stimulant that the trainee might need like tea, coffee, or sweets must be present. They are allowed to smoke, but may have only two drinks per day and only in the evening after the end of course.

It is important that the trainee spends the night at the place of the course, and that he does not have any substantial or exacting contact with the world outside during the course. Naturally the trainee is allowed to call husband/wife or children to tell them that he/she is all right, which is usually not possible in the middle of the week, if the course runs from Monday to Friday. In the holistic healing processes, old emotional pains from your past are confronted. This means that it is very important for the individual to give himself the permission to let go of family and friends for a while, and go back in time, while the process is taking place.

The following steps are considered essential for a successful course:

- **Sale and information** — right from the start the trainee must get the right expectation of what is going to happen. The trainee must understand that it is about *intensive personal development* with the purpose to improve his/her quality of life, health, and ability to function.
- **Examination** — only those that are found to have the sufficient personal resources can be admitted into the course, since it is hard for a participant not to be able to complete the course, which can also disturb the healing process for the other participants. An unfinished process can cause emotional problems for months after the course for the participant. The process can, of course, be finished individually in the holistic medical clinic, but this will cost an additional amount of time, money, and maybe even temporary incapacity from work.
- **Preparation** —the trainee must be informed in writing about the conditions for participating in the course and the contracts the participation implies.
- **Reception** — the trainees must be welcomed in a friendly and positive way. Relaxed talking and behavior will create a pleasurable, sincere, and easy-going sentiment. From the start, the teachers should give their love and support to the participants, and by their example encourage them to "let their hair down", relax, and get ready to be natural, real, and honest for a week.
- **Contracting** — at the beginning of the course the following promises are given:
  - Confidentiality —a holy oath of confidentiality will be taken in order to make everybody feel safe and able to reveal themselves fully.
  - Goodness — to choose to be good. Whenever there is a choice between good and evil, good will be the choice.
  - Faithfulness and loyalty — to be readily available to the partner day and night.
  - Sexual abstention — as long as the course is taking place. Sexual motives can easily dominate above other personal development motives and can dramatically minimize the outcome of the course.
  - Self-discipline and cooperation — to do their homework and exercises.
- **Choice of partner** —it is essential that the trainees work together in pairs. The partner has to be someone that the trainee cares about and that he/she can get on well together with.
- **Training** — in the ability of holding: awareness, respect, care, acknowledgment, and acceptance.
- **Objective** — it is extremely important to make the trainee formulate his/her objective for the course and make an obligation to do the best to solve existential problems and reach the goal of a life with a higher quality of life. If the contract is followed, the goal will most certainly be reached, in spite of how big, incalculable, and impossible it may seem. There is no existential problem that cannot be solved in 5 days if you give what it takes. Every problem that you are able to formulate and realize is ready to be solved. The therapist must believe that the problems are actually always in the consciousness of the participants, which therefore in principle can be changed very fast, if the participants are willing to let go of their negative beliefs.
- **Holistic healing processes** can be controlled, but more often they happen spontaneously. They happen while working with the partner, or though working in plenum in the group. Intensive processes often take place under the daily sharing, when the trainees are sharing their experiences and learning from each other. During the philosophy of life teaching, where many existential concepts and subjects are evaluated[21], a lot often happens with people's personal understanding and attitudes. The most important processes usually happen during the breaks and during social intercourses in the evening, when people open up their hearts, minds, and souls to each other and realize that they truly are meant to give and contribute, not only to receive.
- **Halfway evaluation** is important to measure and thereby correct the objectives continually.
- **Anchorage** — to maintain the process of personal development after the course is of crucial importance. The trainee must realize that the goal for the course has actually been reached and time has come to set other goals. The daily formulation of the goals of the personal development process is highly important.

- **Life status** — Where are you now and where are you going with your life? The development continues and the direction is essentially important. Global quality of life is a central concept[32].
- **Away from the course** — a successful course provides a unique loving and safe environment, where you experience the feeling of being in a good family, maybe for the first time. It is often extremely painful for the trainee to take leave of the other trainees and return to the life that reflects the condition before the course, with a new realization of what a true and good life contains.
- **Networking and progression-groups** — Lists of addresses are given to those that write their names on the lists to support the transformation and to make sure of the gains of the course and a continuation of personal development. Spontaneous establishment of progression groups, where groups of trainees meet after the course and continue their development work, has great value. Often people make friends for life in the courses and sometimes they also find a partner.
- **Process days** — it is a good idea to offer some follow-up days for the trainees to continue to work with themselves, guided by competent holistic therapists (optimally the teachers from the course).
- **The next course** — four continuing week courses are present in our concept, so that people can continue the development they have started. In this way, people will recognize that not everything has to be done tomorrow, and that personal development and improvement of the quality of life, health, and functional ability is a process that takes years and requires a continual personal effort. The first course is called “Introduction to Personal Development”, the second “Personal Transformation”, the third “Find Your Purpose of Life”, and the fourth “Live Your Purpose of Life”. It might be that four courses of 5-days duration, during four consecutive years is not the most efficient series of courses; more research is needed on the most efficient structure of the intervention.

### The Phases of Personal Development

According to the four courses, we have identified four phases of personal development:

1. **Philosophical phase**. In this phase, the trainee is changing from disorientated to orientated about what personal development of quality of life, health, and functional ability is all about. When the trainee has understood the principles behind the holistic process of healing, the next phase begins. In the first phase, the participants often only obtain a few hours of holistic process, and come to let go of one negative decision.
2. **Process phase**. When the mind of the trainee is in accordance with the healing process, and an intention for personal development is combined with personal resources, then holding is the only thing that is needed to make the process continue. In course two, most trainees undergo 20 hours of process, and release 20 substantial negative decisions from a corresponding number of gestalts.
3. **Recognition phase**. The trainee usually experiences the recognition of the purpose of life, which typically takes place at our third course as an essential therapeutic breakthrough. To know who you truly are and discover your purpose with life is an indescribable experience, if you have not been in contact with yourself at a true and deep level before.
4. **Revision of life phase**. “To get a life” is often equivalent with a substantial effort to renew all personal relationships and relations. It might be a year-long project, which demands development of the large talent that is connected with one’s personal purpose of life. Only when you live this talent in all the aspects of life can you be truly happy. Together with happiness comes health and functional ability. Your new life costs you the old life. The transformation is often just as painful as it is necessary, and the objective is about changing from the position of the ego to the position of the true ego[12].

Personal development has the inner logic that you constantly get to mobilize some hidden resources. This makes you capable of integrating some more resource-demanding traumas, which again will release some more of your personal energy. People who are in an intensive developmental progress will therefore often feel that they live on a roller coaster, where mood and energy are altering rapidly and unpredictably. Interestingly, it is possible to open and close the process fairly accurately, so that people are processing from Monday to Friday. Often they can start work again Monday morning after the course, even though some will be a little groggy and dizzy up to 14 days afterwards.

### Some Holistic Group Settings

- **Sharing** — People are sharing what is happening with them, and everybody is learning from that.
- **Small individual processes in plenum** — the trainees are worked with one by one, and the objective of the course is formulated. Existential problems are made clear.
- **Big individual processes in plenum (on the floor with massive holding)** — Whenever there is an excess of resources, even the most far-reaching holistic healing processes can happen quickly, effectively, and painlessly. When a great number of trainees are contributing to the holding, a very intense feeling is reached, which facilitates the process with accelerated speed. Besides contributing to the therapeutic product, the accelerated path is important from an educational point of view, since all the trainees understand how personal development and healing is not just a matter of time, but also a question of intention, holding, and resources.

### Group Therapeutic Tools

- **Give presents.** The most important tool is to give presents generously to the trainees. This is the form that the holding will take when the five qualities are successfully combined. One example is to recognize all the constructive, good, and valuable, which are to be found in them and seen from the outside. It can be especially valuable and call back many hidden resources in the trainee if the giving of presents among the trainees is facilitated. This can be done, for example, if the trainees sit in a circle and in turn receive presents from each other, until each trainee recognizes his own positive nature as a human. Along the process, the trainee typically realizes that the great thing is not to receive, but to give, from exactly that place in your self where you are mostly bright and full of talent. This exercise continues in pairs, whereby the trainees gradually develop themselves and become more and more generous, and less and less critical, demanding, and controlling.
- **Perspective setting.** The most elementary tool is perspective setting. Whenever a constructive, conscious, and loving developing perspective is placed on life, this will often invite the trainee to go into process. The perspective setting must occur in an imaginative and creative way and must adjust to the needs of the trainee. If the trainee has escaped far away from his body to a point in outer space, the therapist has to be imaginative and dynamic enough to meet the patient where he or she is present right now, so that they together can achieve the necessary shift in perspective in a language that the trainee understands. It will be an advantage for the trainee if the therapist is knowledgeable and has several languages, in which he can speak and understand personal development. Some times it is necessary to let go of every reasonable sense in order to help your stuck client. For example you can go on an “astral expedition” with the trainee, who understands himself and his problems in a theosophical reference frame. Under the perspective setting the therapist will use his ability to stay focused.
- **Hyperventilation and physical exercise.** When the trainees need to get in contact with their feelings, you can dance and move about to music. If the contact with the emotions causes particular difficulties for one of the trainees, he can hyperventilate under massive holding, whereby he breathes as powerfully as he can. If this implementation succeeds, the trainee, who is usually a man, will break through in a few hours and be able to feel his emotions and what they contain.

- **Timeline therapy.** A classic timeline therapy is effective whenever the trainee is working with a particular emotion that has roots back to the very early time of his personal history. When people find a final wording for their personal purposes of life, they can often move through the timeline from conception to the present in one step. Mostly, when the participant does this, his life comes to look as one huge event in which the sad theme is: failing my purpose of life. This exercise gives a strange experience of understanding your life completely. In the end of course three, most of the trainees can do this exercise after simple directions. Every time his life is reviewed, new themes and details are recognized. This is a typical exercise in awareness and recognition.
- **Encounter and reflection.** The technique exists in so many different versions, some of which are extremely primitive, almost violent, and has given the concept of therapeutic encounter a negative image. For example, the trainee and the therapist can hit each other by turns until the trainee understands the nature of his oppressed anger, and finds negative attitudes and decisions to let go of. An example of a more modern use of the encounter technique is a quiet confrontation face to face with the intention of solving a problem, in the form of a difficult life theme for the person. The secret lies in supporting the trainee to search himself into the difficult field, where the center almost every time is an unbearable, emotional pain from their personal history. This process can be used to help the trainee stop being deliberately evil[15]. This particular exercise carries the popular name “devil exorcise”, not because it contains any belief in Satan, but because of its superficial resemblance with a corresponding technical process done by Christian ministers. The participant appears existentially divided, because he or she insists on being good and evil at the same time. The good comes from the contact with his true nature, which is always good, and the evil comes from the empirical experience of evilness in an evil environment. In the session, the trainee is asked to be as evil as he can be, whereafter the therapist confronts this evilness and hereby helps the trainee to clearly see what it is all about. As the dark side becomes clearer, it becomes more and more untenable, because the white side of the trainee — his true self — is allied with the therapist. In the end, the black side fragments in a series of memory-pictures of painful experiences with corresponding negative decisions. When the trainee lets go of the repressed traumas from the personal history and rediscovers precise formulations of his negative decisions from the gestalts, all the unconscious evil intentions that buzzed up to the evil personality will disappear, much like Dr. Jekyll’s “Mr. Hyde”[33]. Another version of encounter is mirroring, where the therapist helps the trainee to discover a positive or negative aspect of his own personality.
- **Exercises in pairs to support lovingly, to communicate respectfully, and to take your projections back.** The partners work their way through the course by giving each other holding. The pairing happens as the trainees’ walk around each other until they find a partner, and sometimes the teachers assist the process. Because the choice of partner happens this way, there is going to be a lot in common between the pairs. This leads to massive projections that must be handled through awareness. It is far easier to discover mistakes with the other than with your self. This process is often very productive and full of learning. Otherwise, the principle is that you are responsible for working with your own themes, and the partner supports you as well as he or she can without interfering. Honest and respectful communication is ingrained at the beginning of the course to ensure that the communication remains honest and takes on a constructive and good character. The pairs reflect each other’s neuroses and help each other to an awareness of all the coincidences of “can’t have” and “must have” that historical life pains have left through negative decisions. Understanding of the fundamental cause of the problems, symptoms and diseases is a very important part of the developing process.

### The Qualifications of the Therapist

Certain qualifications are needed to lead a group session in existential holistic group therapy working with the principles of holistic process theory. Besides the support that lies in being a health professional like a physician or a nurse, it is necessary to have a thorough knowledge of the underlying theories and to master quality of life philosophy and a positive philosophy of life in general. It is necessary to be qualified as a holder, which means that you have to be able to give the trainees neutral attention, respect, loving care, acknowledgment, and acceptance. It is not realistic to be able to give all these qualities to everybody. But you need to give correct holding to the persons you choose to help.

It is also the function of the teachers to back off from trainees who cannot be loved and held as they are. This is the fundamental precondition for the trainee to get a good result and have the opportunity to enter the holistic healing process. The sexual limits of the therapist must be respected, since great transference and several intimate contacts otherwise might lead to a violation of the trainees. The most important qualification of the therapist is obviously a thorough comprehension of himself and his own mission, which must be in accordance with the purpose of the course. When someone recognizes himself, it is easier for him to recognize other people. A therapist, who has a good insight into his own positive and negative parts, will be able to comprehend the trainee in a sharp and precise way that often surprises.

The functions of process-operator, holders, and practical assistants must be correctly distributed so that there exists one holder per 15 trainees, and enough assistants to take care of all the practical work without any difficulties. All the participants that are personnel must deeply understand what is happening at the course and share the intention with the course, so that they will not exist as a brake for the process of the trainees.

### Coherence in the Group and Collective, Intuitive Knowledge

It is possible for a trained holistic therapist to know intuitively which gestalts the trainee is working with. The details are often quite surprising, as it is possible intuitively to know at which age — often in childhood — a gestalt originated, and the emotional content of it. The skilled existential holistic therapist can often tell what happened in the painful event, and even know the formulation of the negative decision that was made before the trainee knew it. The intuitive knowledge seems to be a function of the strength of the coherence in the group; this coherence seems to arise when the therapist and the trainees are connecting emotionally and even spiritually (i.e., connected through to love and purpose of life[3]). If everybody is present in the three dimensions of existence (purpose, consciousness, and gender, see theory of talent[13]), it seems that everybody can share the same feelings and that the intuitive knowledge of the group members becomes collective knowledge. This is fairly mysterious, related to the famous and controversial concept of the collective subconsciousness of Carl Gustav Jung[16]; it might be a good example of sharing biological information, much in the same impressive way as a colony of ants, corals, and other social animals do[24,25,26].

## RESULTS

Spontaneous remissions of chronic pain and other illnesses seem to happen far more effectively in a group setting, where all the participants stand together and support each other, than in the clinic, where the therapist is alone with the patient. A 5-day course in personal development can be compatible to a half year of individual holistic therapy sessions[19,20,21]. In two pilot studies, we found the effect of such a 5-day course to be a 10% improvement of global quality of life and self-assessed health, and for patients with chronic pains we found a 40% reduction of the pain[19,20]. Radical improvements of all aspects of life — physical, mental, sexual, social, and spiritual — were reported[21]. A small percentage of the patients showed incidents of psychoses shortly after the course, but interviews of these patients together with a psychiatrist did not show any lasting problems or side effects. The patients reported that in spite of these unpleasant episodes, their quality of life and subjective health had improved in general, because of the learning from the existential holistic group therapy. From the pilot studies, we believe today that two-third of the trainees are helped by the courses, but more research is needed.

### Side Effects of Existential Holistic Group Therapy

The holistic processes of healing will bring the trainee in contact with earlier incidents, often with a very high — and as things move forward, increasing — intensity of emotional pain. The development process can be set on hold if the trainee, on his soul level, does not want to let go of his old self and develop himself. This is often incomprehensible for the trainee, because a clearly formulated mental desire exists to move on with one self and make life better. There is a risk that this patient will continue being “in process” after the course, which the therapist cannot do much about; individual therapy will be needed for the trainee the following month. Existential pains that are contacted without the trainee taking responsibility for them can bring the trainee on the way to psychosis or even further, especially if there have been psychotic episodes earlier in life; it is very important that the trainee knows this and agrees beforehand to contact his psychiatrist if severe mental problems appears after the course.

Frequently in therapy, events can be found where the parents have driven the trainee almost crazy in early childhood, without the parents being aware of the serious condition of the child. Gestalts with psychotic contents are processed like any other gestalts; what makes the content psychotic is the character of the decisions that human took to escape from the existential pain in the situation: “nothing exists”, “you are dead” or “I do not exist”. Often it is exactly the decisions with de-personalizing, de-realization, and de-vitalization, which makes the trainee unwilling to move further into process. Experience has shown, that some trainees can feel unpleasant for several months, while they gradually return to their condition prior to the course.

As the side effects are severe for a few of the trainees, we recommend that courses in existential holistic group therapy be lead by a physician. It is very important to note that the most sick patients experience the most severe side effects, and that the observed progress generally has been larger the more sick a person is. After 4 years of work with existential holistic group therapy we find the side effects very limited compared to the therapeutic effects.

### Assessment Before and After the Course

A long period with poor quality of life and perhaps even incapacity for work is a very serious side effect of existential group therapy. This can however frequently be avoided with a thorough assessment before starting. This assessment must assure that people who do not have a real intention of developing themselves are not allowed in the course. Patients with violent, criminal, psychiatric history or patients who take psychotropic drugs should have a very thorough assessment. If they are accepted for the course, a psychiatric follow-up or even, with high-risk patients, a psychiatric residence must be prepared for them afterwards. Often it will be necessary to have one or two holders connected to one psychiatric patient. The therapy can often be very useful to the psychiatric patient and help the patient to get out of his psychiatric disease. Therefore we do not think that psychiatric patients should be barred outright from existential holistic group therapy.

A suspicion about suicidal tendencies should lead to the decision that the trainee is not accepted at the course. Suicidal thoughts during or after the course should involve obligatory psychiatric supervision, but practically there are seldom difficulties as long as the assessment or initial screening is done properly. A small global quality of life questionnaire like QOL5[34] can be used for screening the trainees for severe problems, along with a personal confrontation before starting the course.

Quality control and quality improvement can be done according to the square curve paradigm[35] if you measure quality of life and health with QOL5 or a similar questionnaire before and after the intervention, and one year after; such a method for evaluation will clearly show how much the group as a whole and its individual members have been helped, and is of course strongly recommended. In this way the effect of the therapy can also be scientifically documented, even if the trainees consist of an inhomogeneous group with regard to diagnoses, the conditions that motivated them for holistic therapy, and personal development.

Interestingly, existential holistic group therapy seems to benefit almost everybody who wants to heal from the bottom of his or her existence. If the group consists of normal people not being diagnosed, but motivated solely by the need of a personal upgrade of quality of life and performance, it is of equal importance to document the effect and evaluate the results.

### CONCLUSION

Massive holding combined with personal resources and an intention to heal means that old wounds on the soul can heal completely, even surprisingly quickly and painlessly in the resource-full environment that the well-functioning group creates for the individual. Existential group therapy leads the trainee or the patient back to the most painful and difficult incidents in his/her life. The holistic healing process takes form in the group as laughter and tears (step 1: feel), shifting between deep reflection (step 2: understand), changing into enthusiasm about self-insight, and new perspectives (step 3: let go).

The qualifications of the therapist, theoretically as well as practically, and an initial screening or assessment of the participants with a suitable environment for the course are important components for the success of the course. It is essential to have a contract at the beginning of the course in order for the participant or trainee to set high goals from the start. The trainees themselves do most of the work and it is their own responsibility that they reach some results. A generous environment, where it is more important to give than to receive, where it is acceptable to fail even though you are doing your best, and where everybody is learning from everything that happens, characterizes a well-functioning existential holistic therapy group.

Spontaneous healing happens far more effectively in a group setting, where all the participants stand together and support each other, than in the clinic, where the therapist is alone with the patient. A 5-day course in personal development can be compatible to a half year of individual holistic therapy sessions, and radical improvements of all aspects life and quality of life: physical, mental, sexual, social, and spiritual, have been reported.

### ACKNOWLEDGMENTS

This study was supported by grants from The 1991 Pharmacy Foundation, as well as by supplementary grants from Goodwill-fonden, the JL-Foundation, E. Danielsen and Wife's Foundation, Emmerick Meyer's Trust, the Frimodt-Heineken Foundation, the Hede Nielsen Family Foundation, Petrus Andersens Fond, Wholesaler C.P. Frederiksens Study Trust, Else & Mogens Wedell-Wedellsborg's Foundation and IMK Almene Fond. The research was approved by the Copenhagen Scientific Ethical Committee under number (KF)V.100.2123/91.

### BIOSKETCHES

**Søren Ventegodt, MD,** is the Director of the Quality of Life Research Center in Copenhagen, Denmark. He is also responsible for a Research Clinic for Holistic Medicine in Copenhagen and is a popular speaker throughout Scandinavia. He has published numerous scientific or popular articles and a number of books on holistic medicine, quality of life, and quality of working life. His most important scientific contributions are the comprehensive SEQOL questionnaire, the very short QoL5 questionnaire, the integrated QOL theory, the holistic process theory, the life mission theory, and the Danish Quality of Life Research Survey, 1991–94 in cooperation with the University Hospital of Copenhagen and the late pediatric professor Bengt Zachau-Christiansen. E-mail: ventegodt@livskvalitet.org. Website: www.livskvalitet.org/

**Niels Jørgen Andersen, MSc,** Professor, Department of Innovation and Economic Organization, Norwegian School of Management. This department conducts research and provides teaching in central topics related to innovation, business development, management of global companies, business history, and economic organization. Research activities within the Department are related to four core subjects within the discipline: business history, cooperative organizations, business development and entrepreneurship, and finally studies of industries with a special focus on the electricity industry. He is also the dynamic chairman of the nonprofit organization Stiftelsen Holistisk Medisin Scandinavia, which aims to support the scientific development, research, and documentation of complementary and holistic medicine in Scandinavia. E-mail: niels.j.andersen@bi.no. Website: www.bi.no/users/fgl93013/

**Joav Merrick, MD, DMSc,** is Professor of Child Health and Human Development affiliated with the Zusman Child Development Center, Division of Pediatrics and Community Health at the Ben Gurion University, Beer-Sheva, Israel; the Medical Director of the Division for Mental Retardation, Ministry of Social Affairs, Jerusalem; and the Founder and Director of the National Institute of Child Health and Human Development. He has numerous publications in the field of child and human development, rehabilitation, intellectual disability, disability, health, welfare, abuse, advocacy and prevention. Dr. Merrick received the Peter Sabroe Child Award for outstanding work on behalf of Danish Children in 1985 and the International LEGO-Prize ("The Children's Nobel Prize") for an extraordinary contribution towards improvement in child welfare and well being in 1987. E-mail: jmerrick@internet-zahav.net. Website: www.nichd-israel.com
